# Home and Online Management and Evaluation of Blood Pressure (HOME BP) using a digital intervention in poorly controlled hypertension: randomised controlled trial

**DOI:** 10.1136/bmj.m4858

**Published:** 2021-01-19

**Authors:** Richard J McManus, Paul Little, Beth Stuart, Katherine Morton, James Raftery, Jo Kelly, Katherine Bradbury, Jin Zhang, Shihua Zhu, Elizabeth Murray, Carl R May, Frances S Mair, Susan Michie, Peter Smith, Rebecca Band, Emma Ogburn, Julie Allen, Cathy Rice, Jacqui Nuttall, Bryan Williams, Lucy Yardley, HOME BP investigators, Adam Jones, Ajmal Hussain, Alistair McHardy, Anna Harrison, Anna LaLonde, Asim Malik, Basilio Hernandez-Diaz, Ben Cranfield, Brian Nicholson, Carl Anandan, Catherine Neden, Cathy Bobrow, Chloe Evans, Christopher Keast, Clare Henderson, Corrine Sutherland, Craig Kyte, Dan Henderson, Debbie Noble, Ed Capo-Bianco, Edward Williams, Elizabeth Shaw, Fatima Mohri, Gaurav Asal, Greig Dougall, Hardeep Bhupal, Heidi Luckhurst, Hergeven Dosanjh, Hilary Nowell, Jan Brown, Jennifer Flett, Julian Barber, Heather Rutter, Julian Thompson, Kanjhana Ramanan, Karen Madronal, Karen Malone, Katie Etherington, Kenney Tsoi, Kyle Knox, Laila Amin, Lisa Hirst, Lucy Allen, Luke Skellern, Lynne Flynn, Lorne McEwan, Mark Pugsley, Marloes Frassen, Matthew Gaw, Matthew Prendergast, Matthew Wallard, Muhammad Faisal, Nick Wooding, Nienke Lees, Paul Wainman, Nithya Nanda, Patrick Moore, Paul Conley, Paul Johnson, Penny Wilson, Phillip Jacobs, Pippa Whitbred, Rebecca Zamir, Richard Reed, Richard Tribley, Richard Woof, Ruth Danson, Ruth Lawes, Sarah Gallagher, Sarah Wadsworth, Serge Macanovic, Simon Cartwright, Simon Pettitt, Simon Tucker, Stephen Doggett, Tamsin Sevenoaks, Tara Watson, Tess Talbot, Ruth Imrie, Thomas Herbert, Tony Crockett, Tony Wright, Vanshika Sharma, Vicki Telford, Zaid Almashta, Zelda Cheng, Zishan Ali, Alice Grube, Andy Claxton, Barbara Asante, Becki Weare, Eleanor McKee, Bertha Werrett, Carmel Barwell, Carole Mulvihill, Caroline Sherwood, Clare MacDonald, Dadirayi Tabor, Dawn Denning, Debbie Roberts, Diane Adshead, Gemma Clarke, Heather Huntley, Heather Pinder, Irene Qasim, Jane Merrison, Jill King, Julie Allison, Kam Johal, Karen Terry, Karine Wood, Kathryn Balmford, Katie Barnes, Katie Post, Kelly-Marie Bowden, Kirsty Edmunds, Klaire Whittle, Lara Peniket, Leann Carnegie, Linda Neale, Lisa Davey, Liz Bartlett, Louise Smith, Lucy Clack, Martina Brown, Naomi McKenna, Pam Kay, Polly Jacobs, Rebecca Cutts, Robert Pearse, Ruth Atkinson, Sally Barter, Sally Mackie, Sam Norris, Sandra Cook, Sarah Elderfield, Sarah Nzante, Sharon Cronin, Sophie Maslen, Stephanie Marchant, Stephanie Wright, Sue Keene, Sue Smith, Suzie Cimelli, Tracy Stone, Tricia Joyce, Trudie Le Marechal, Vicky Kettle, Victoria Osborne, Wendy Cubiss, Wendy Marsden, Wioletta Kowalczyk-Williams, Zoe Bailey

**Affiliations:** 1 Nuffield Department of Primary Care Health Sciences, University of Oxford, Oxford OX2 6GG, UK; 2 Primary Care and Population Sciences Unit, University of Southampton, Southampton, UK; 3 School of Psychology, University of Southampton, Southampton, UK; 4 eHealth Unit, Research Department of Primary Care and Population Health Sciences, University College London, London, UK; 5 Faculty of Public Health and Policy, London School of Hygiene and Tropical Medicine, London, UK; 6 General Practice and Primary Care, Institute of Health and Wellbeing, University of Glasgow, Glasgow, UK; 7 UCL Centre for Behaviour Change, University College London, London, UK; 8 Patient and Public Contributor, Bristol, UK; 9 Southampton Clinical Trials Unit, University of Southampton, Southampton, UK; 10 Institute of Cardiovascular Sciences, NIHR UCL Hospitals Biomedical Research Centre, University College London, London, UK; 11 School of Psychological Science, University of Bristol, UK

## Abstract

**Objective:**

The HOME BP (Home and Online Management and Evaluation of Blood Pressure) trial aimed to test a digital intervention for hypertension management in primary care by combining self-monitoring of blood pressure with guided self-management.

**Design:**

Unmasked randomised controlled trial with automated ascertainment of primary endpoint.

**Setting:**

76 general practices in the United Kingdom.

**Participants:**

622 people with treated but poorly controlled hypertension (>140/90 mm Hg) and access to the internet.

**Interventions:**

Participants were randomised by using a minimisation algorithm to self-monitoring of blood pressure with a digital intervention (305 participants) or usual care (routine hypertension care, with appointments and drug changes made at the discretion of the general practitioner; 317 participants). The digital intervention provided feedback of blood pressure results to patients and professionals with optional lifestyle advice and motivational support. Target blood pressure for hypertension, diabetes, and people aged 80 or older followed UK national guidelines.

**Main outcome measures:**

The primary outcome was the difference in systolic blood pressure (mean of second and third readings) after one year, adjusted for baseline blood pressure, blood pressure target, age, and practice, with multiple imputation for missing values.

**Results:**

After one year, data were available from 552 participants (88.6%) with imputation for the remaining 70 participants (11.4%). Mean blood pressure dropped from 151.7/86.4 to 138.4/80.2 mm Hg in the intervention group and from 151.6/85.3 to 141.8/79.8 mm Hg in the usual care group, giving a mean difference in systolic blood pressure of −3.4 mm Hg (95% confidence interval −6.1 to −0.8 mm Hg) and a mean difference in diastolic blood pressure of −0.5 mm Hg (−1.9 to 0.9 mm Hg). Results were comparable in the complete case analysis and adverse effects were similar between groups. Within trial costs showed an incremental cost effectiveness ratio of £11 ($15, €12; 95% confidence interval £6 to £29) per mm Hg reduction.

**Conclusions:**

The HOME BP digital intervention for the management of hypertension by using self-monitored blood pressure led to better control of systolic blood pressure after one year than usual care, with low incremental costs. Implementation in primary care will require integration into clinical workflows and consideration of people who are digitally excluded.

**Trial registration:**

ISRCTN13790648.

## Introduction

Hypertension is the major risk factor for cardiovascular disease internationally and evidence from several randomised controlled trials shows that this risk can be reduced by lowering blood pressure.^1^ ^2^ In the United Kingdom, almost 30% of adults have raised blood pressure (≥140/90 mm Hg), with the proportion increasing to more than 50% in people aged 65 and older.^3^ Target blood pressure levels are reached for less than half of adults, and with an ageing population, novel interventions are required to improve blood pressure control.^3^ ^4^

Digital interventions (apps, programmes, or software used in a health context) have the potential to support people in self-management.^4^ ^5^ A digital intervention developed by our group that facilitates lifestyle change in primary care patients with obesity resulted in cost effective weight loss.^6^ However, for patients with hypertension, evidence for digital interventions has been from small trials with relatively short follow-up and substantial heterogeneity of results.^7^ One trial that lasted longer than 10 months reported that patients’ blood pressure was not reduced.^8^ We have previously shown that self-monitoring combined with self-titration of antihypertensive drugs is cost effective and leads to considerably lower blood pressure in people with essential hypertension and those at higher risk. However, this approach requires extensive manual record keeping, which makes implementation difficult.^9^ ^10^ ^11^ ^12^ Therefore, a digital intervention that combines our previous knowledge of self-management of hypertension with digital support and lifestyle changes might result in lower blood pressure with associated lifestyle changes, including weight management. This intervention could be more easily integrated into clinical care by patients and healthcare professionals, and would allow remote monitoring, an important consideration when people are restricted to staying home because of disability or to avoid exposure to infection. Clinical monitoring of these patients would be difficult or impossible.^13^

The HOME BP trial aimed to evaluate whether a digital intervention comprising self-monitoring of blood pressure with reminders and predetermined drug changes combined with lifestyle change support resulted in lower systolic blood pressure in people receiving treatment for hypertension that was poorly controlled, and whether this approach was cost effective.

## Methods

The methods of the HOME BP trial have already been described in detail.^14^ Briefly, it was an unmasked randomised controlled trial with automated ascertainment of outcome. The study compared a digital intervention for hypertension management and self-monitoring of blood pressure with usual care (routine hypertension care, with appointments and drug changes made at the discretion of the general practitioner).

### Population

Eligible participants were aged 18 or older with treated hypertension, a mean baseline blood pressure reading (calculated from the second and third blood pressure readings) of more than 140/90 mm Hg, and were taking no more than three antihypertensive drugs. For the digital intervention, participants needed to be willing to self-monitor and have access to the internet (with support from a family member if needed).

Exclusions included blood pressure greater than 180/110 mm Hg, atrial fibrillation, hypertension not managed by their general practitioner, chronic kidney disease stage 4-5, postural hypotension (>20 mm Hg systolic drop), an acute cardiovascular event in the previous three months, terminal disease, or another condition which in the opinion of their general practitioner made participation inappropriate.

### Procedure

We used practices from the National Institute for Health Research Clinical Research Network (https://www.nihr.ac.uk/explore-nihr/support/clinical-research-network.htm). Eligible people were identified from clinical codes recorded in the electronic health records of collaborating general practices; these people were invited to attend a baseline clinic to learn about the study. At the clinic, eligibility was established, informed consent was obtained from people who wished to participate, and their baseline clinical data were collected. People who did not want to take part in the trial were given the option to complete a form giving their reasons. Study nurses measured participants’ blood pressure after five minutes’ rest using a standardised technique with a validated electronic automated sphygmomanometer (BP TRU BPM 200).^15^ Six blood pressure readings were taken at intervals of one minute. All participants completed online questionnaires.

Eligible participants were randomised using an online system (https://www.lifeguideonline.org) in a 1:1 ratio to receive usual care or the HOME BP intervention with optional nurse support. Minimisation factors were participants’ baseline systolic blood pressure, age, diabetes status, and practice. Practice staff were notified of patient group allocation by email.

After randomisation, all patients received a blood pressure drug review from a general practitioner or nurse prescriber (prescribers). For patients allocated to the intervention group, prescribers were asked to select and agree an individualised drug titration plan (including three potential drug changes if blood pressure remained above target).^10^ Participating clinicians were given information about the National Institute for Health and Care Excellence (NICE) guidance within the HOME BP intervention but were not asked to follow set algorithms for drugs.

Six and 12 months after randomisation, participants attended follow-up appointments with an independent research nurse where blood pressure and weight were recorded (weight was recorded at 12 months only). Participants then completed follow-up questionnaires online.

### Intervention

The HOME BP intervention for the self-management of high blood pressure consisted of an integrated patient and healthcare practitioner online digital intervention, blood pressure self-monitoring, healthcare practitioner directed and supervised titration of antihypertensive drugs, and user selected lifestyle modifications. The intervention was developed using a theory, evidence, and person based approach^16^ that was designed to influence the behaviour of participants and healthcare professionals. The development process has been fully described elsewhere; briefly, it comprised an iterative process including think aloud interviews to provide detailed feedback on the HOME BP prototype.^17^ ^18^ ^19^

Participants were given online instructions on how to correctly undertake self-monitoring (using an Omron M3 monitor), with a demonstration video.^20^ Participants were then asked to rehearse self-monitoring for a minimum of seven days and enter these initial readings into HOME BP online before undertaking study procedures.

Participants were advised, with automated email reminders, to take two morning blood pressure readings for seven days each month and to enter online each second reading (following methods used in the TASMINH2 and TASMIN-SR studies).^21^ ^22^ Mean home blood pressure was then calculated and feedback provided to the participants and the healthcare practitioners by using a traffic light system (developed from the system used in previous drug titration procedures).^21^ ^22^ When mean home blood pressure was above target for two consecutive months, the prescriber was asked by email to implement the preplanned drug change.

The following home blood pressure targets were set in line with up-to-date NICE hypertension guidelines^23^; an adjustment of 5/5 mm Hg was made for home readings^24^:

- People younger than 80 without diabetes: less than 135/85 mm Hg
- People aged 80 and over without diabetes: less than 145/85 mm Hg
- People with diabetes: less than 135/75 mm Hg.

When home blood pressure readings were very high (>180/110 mm Hg) or very low (systolic blood pressure <100 mm Hg), patients were advised to call their general practitioner within three days and HOME BP healthcare practitioners were alerted by email. If mean home blood pressure was controlled for three consecutive months (defined as 100-134/≤84 mm Hg), patients were advised to reduce blood pressure monitoring to once every eight weeks; monitoring reverted back to every month if mean blood pressure subsequently increased above 135/85 mm Hg.

The HOME BP intervention included elements designed to motivate and support healthy behaviours. Information was presented about the health related benefits of self-monitoring, about reducing blood pressure through drugs, and addressing common patient concerns about the side effects of drugs. Nine weeks after participants were allocated to the intervention (judged to be sufficient time for self-monitoring habits to have been implemented), an optional tool became available outlining user selected evidence based lifestyle modifications that target healthy eating, physical activity, losing weight (if appropriate), and salt and alcohol reduction.^14^ The health behaviours targeted were chosen based on normalisation process theory and took the form of web pages and links.^17^

The HOME BP intervention also aimed to build healthcare practitioner motivation, knowledge, and skills to reduce clinical inertia.^17^ This objective was achieved by presenting evidence of efficacy and addressing concerns about patient titration acceptance, the reliability of home blood pressure readings, and study procedures.

Optional additional behavioural support for self-monitoring and lifestyle modifications was available to intervention participants through practice nurses or healthcare assistants (referred to as supporters) by using the CARE (congratulate, ask, reassure, encourage) approach.^17^ This support comprised up to six brief face-to-face, telephone, or email contacts addressing difficulties associated with self-monitoring or lifestyle change, with additional monthly email support provided by using prewritten templates.

### Usual care

Participants allocated to usual care were not provided with self-monitoring equipment or the HOME BP intervention, but had online access to the information provided in a patient leaflet for hypertension (patient.co.uk; through the same system that delivered the online questionnaires). This information comprised definitions of hypertension, causes, and brief guidance on treatment, including lifestyle changes and drugs. These participants received routine hypertension care that typically consisted of clinic blood pressure monitoring to titrate drugs, with appointments and drug changes made at the discretion of the general practitioner. Participants were not prevented from self-monitoring; data on self-monitoring practices were collected at the end of the trial from patients and practitioners.

In intervention and usual care groups, decisions about patients’ drugs remained at the prescriber’s discretion at all times.

### Outcomes

The primary outcome of the trial was the difference in clinic systolic blood pressure (mean of second and third readings) at 12 month follow-up between the intervention and usual care groups, adjusting for baseline blood pressure, practice, blood pressure target levels, and sex. Secondary outcomes (also adjusted for baseline and covariates if appropriate) included systolic and diastolic blood pressure at six and 12 months using second and third blood pressure readings, and second to sixth blood pressure readings; weight; modified patient enablement instrument (patients’ feelings of confidence about understanding their illness and their ability to manage, understand, and cope with their condition; and general health problems that occurred after receiving healthcare)^25^ ^26^; drug adherence (Medication Adherence Rating Scale questionnaire)^27^; health related quality of life measured with the EuroQoL-5D-5L^28^; and side effects from the symptoms section of an adjusted illness perceptions questionnaire.^29^

After trial registration, participants and general practitioners were asked about use of self-monitoring in the usual care group. At the end of the trial a medical record review captured prescription of antihypertensive drugs (including any changes) and within trial primary healthcare resource use (primary care and secondary care, including outpatient and inpatient visits). Additional outcomes that will be published elsewhere included long term economic modelling and a detailed process evaluation.

### Power calculation and analysis

A sample size of 244 patients for each group was required to have 90% power to detect a difference in systolic blood pressure of 5 mm Hg (standard deviation 17 mm Hg) between the intervention and usual care groups based on the findings from the TASMINH-2 study.^22^ Allowing for a 15% participant dropout, 287 participants were required for each group, resulting in a total sample size of 574 participants. During the trial, we decided to increase the sample size to 610 to allow for a 20% dropout rate because of concerns about higher than expected initial dropout (which later proved unfounded).

The principal analysis used raw and adjusted data, and was agreed in a statistical analysis plan before final data lock (see appendix). The primary analysis used general linear modelling to compare systolic blood pressure in the intervention and usual care groups at follow-up, adjusting for baseline blood pressure, practice (as a random effect to take into account clustering), blood pressure target levels, and sex. Analyses were on an intention-to-treat basis and used 100 multiple imputations by chained equations for missing data. The imputation model included all outcome and stratification variables. Sensitivity analyses used complete cases and also a repeated measures technique. Planned subgroup analyses included blood pressure target groups, older versus younger participants (67 as threshold), men versus women, lower index of multiple deprivation scores versus higher scores, and blood pressure better controlled at baseline versus worse controlled at baseline (above or below median systolic blood pressure). Secondary analyses used similar techniques to assess differences between groups. Post hoc we decided to present antihypertensive drugs as the number of dose changes and drug changes rather than as defined daily dose (which combines dose and number of drugs) to show the specific type of changes more clearly.

A within trial economic analysis estimated cost per unit reduction in systolic blood pressure—the primary outcome—by using similar adjustments and multiple imputation for missing values as described in the statistical analysis section. UK National Health Service resource use costs included those due to the intervention and those due to changes in drugs and use of other relevant NHS resources. The following items were costed (further details in appendix table A6):

- Antihypertensive drugs (by dose and dose changes, and any new antihypertensive drugs, all by number of days used) from NHS drug tariff 2018^30^
- Primary care contact related to blood pressure (by type of staff—general practitioner, practice nurse, or healthcare assistant) and type of contact (face to face, telephone, email, or text, including clinical contacts from supporting the intervention)^31^
- Inpatient admission (by health resource use code), outpatient visit, or emergency department attendance related to hypertension.^32^

We used repeated (1000 times) bootstrapping to estimate the probability of the intervention being cost effective at different levels of willingness to pay per unit reduction in blood pressure.

### Trial registration and approvals

HOME BP is registered as ISRCTN13790648 (https://doi.org/10.1186/ISRCTN13790648). The original registration was for the development and pilot phase, which ran into the main trial without change. Except for an increase in sample size as documented above, no other substantive changes were made to the protocol after the start of the trial. The trial registration did not specify which blood pressure measurements were to be used in the secondary outcomes, but these were clarified in the statistical analysis plan before data lock as the mean of the second and third measurements, and the mean of the second to sixth measurements. Post hoc analyses are stated.

### Patient and public involvement

Patient and public contributors were involved from the outline application stage (Samantha Hall and Mark Stafford-Watson). At full application stage these contributors were joined by Keith Manship and Shelley Mason. Key aspects contributed to were the development of the intervention, commenting on trial documentation, and taking part in the steering group meetings. Cathy Rice joined as a patient and public contributor during the trial and has remained extensively involved, including optimising many patient facing documents and intervention training content, authorship of this paper and assisting in dissemination. We are immensely grateful for the input of all of our public contributors.

## Results

Of 11 399 invitation letters sent out, 1389 (12%) potential participants from 76 general practices responded positively and were screened for eligibility. Those who declined to take part could optionally give their reasons, and responses were gained from 2426/10 010 (24%). The mean age of those who gave a reason for declining was 73 years. The most commonly selected responses were not having access to the internet (982, 41%), not wanting to be part of a research trial (617, 25%), not wanting to participate in an internet study (543, 22%), and not wanting to change drugs (535, 22%; table A1).

**Fig 1:**
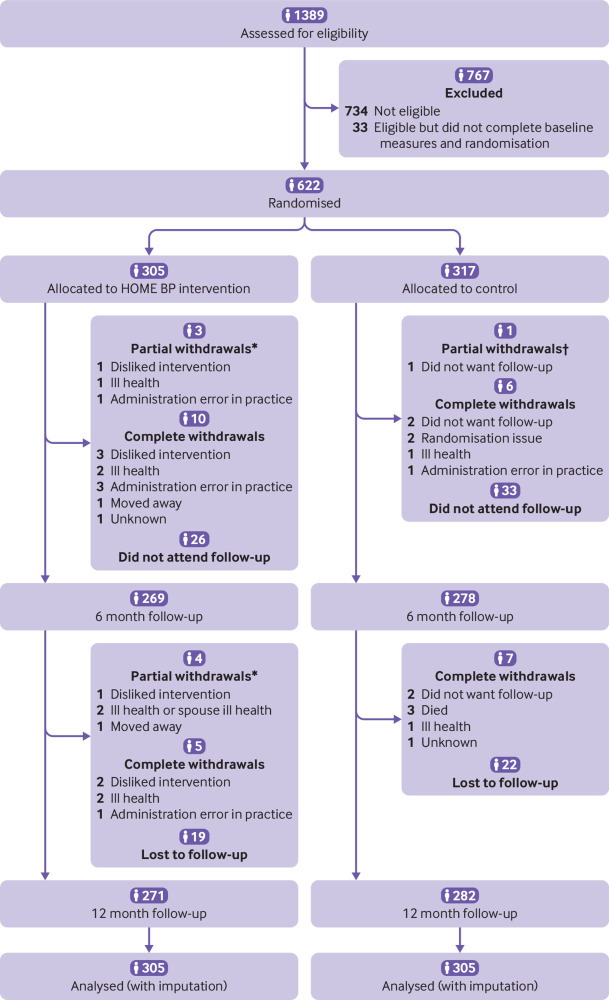
Flowchart of HOME BP (Home and Online Management and Evaluation of Blood Pressure) trial. *Partial withdrawals withdrew from the intervention but consented to be followed up. †Partial withdrawals in usual care consented to passive follow-up

Of the 1389 screened, 734 were ineligible and so were excluded (fig 1). A further 33 did not complete baseline measures and randomisation, which left 622 people who were randomised to the HOME BP intervention or usual care (305 and 317, respectively; proportions are in line with the minimisation algorithm). The main reasons for exclusion (denominator 734 in each case) were blood pressure less than 140/90 mm Hg (652, 89%), postural hypotension (31, 4%), not taking antihypertensive drugs (18, 2%), and blood pressure too high (>180/110 mm Hg; 16, 2%). Fifteen people (2%) who did not fulfil the inclusion criteria because their blood pressure was out of range were randomised in error (in 10 people blood pressure was too high; in five people blood pressure was too low). After we discussed the issue with the sponsor and with the relevant general practitioners, we decided to keep these people in the trial unless they wished to withdraw, and they have been included in the intention-to-treat analysis.

The groups were well matched, with a mean age of 66 years and mean baseline clinical blood pressure of 151.6/85.3 mm Hg and 151.7/86.4 mm Hg (usual care and intervention, respectively; table 1). Most participants were white British (94%), just over half were men, and time since diagnosis averaged around 11 years. The most deprived group accounted for 63/622 (10%), with the least deprived group accounting for 326/622 (52%).

**Table 1:** Baseline characteristics of usual care and intervention groups in the HOME BP trial. Data are numbers (percentages) unless stated otherwise

Baseline characteristics	Usual care (n=317)	Intervention (n=305)
Age, years (mean (standard deviation))	66.7 (10.2)	65.2 (10.3)
Systolic blood pressure (mean (standard deviation))	151.6 (11.1)	151.7 (11.8)
Diastolic blood pressure (mean (standard deviation))	85.3 (9.9)	86.4 (9.6)
Female	143/317 (45.1)	145/305 (47.5)
Ethnicity		
White	299/317 (94.3)	285/304 (93.8)
Black African	3/317 (1.0)	5/304 (1.6)
Black Caribbean	1/317 (0.3)	0/304 (0.0)
Indian	0/317 (0.0)	3/304 (1.0)
Pakistani	3/317 (1.0)	1/304 (0.3)
Other	11/317 (3.5)	10/304 (3.3)
Index of multiple deprivation		
1-3 (most deprived)	27/317 (8.5)	36/304 (11.8)
4-7	124/317 (39.1)	108/304 (35.5)
8-10 (least deprived)	166/317 (52.4)	160/304 (52.6)
Marital status		
Married or cohabiting	244/317 (77.0)	240/302 (79.5)
Single, divorced, or widowed	73/317 (23.0)	62/302 (20.5)
Duration of hypertension, years (mean (standard deviation))	10.9 (9.4)	11.3 (9.8)
Past medical history		
Diabetes	32/291 (11.0)	24/278 (8.6)
Type 1	1/291 (0.3)	1/278 (0.4)
Chronic kidney disease	26/291 (8.9)	22/279 (7.9)
Stroke	3/292 (1.0)	2/278 (0.7)
Myocardial infarction	4/291 (1.4)	7/278 (2.5)
Coronary artery bypass graft, angioplasty, or stent	3/292 (1.0)	10/278 (3.6)
Other comorbid condition	67/288 (23.2)	70/273 (25.6)
Body mass index (mean (standard deviation))	29.6 (5.4)	30.2 (6.6)
No of antihypertensive drugs at baseline (median (interquartile range))	1 (1-2)	1 (1-2)

HOME BP=Home and Online Management and Evaluation of Blood Pressure.

After 12 months, primary endpoint data were available from 271 (89%) participants in the intervention group and 282 (89%) in the usual care group (fig 1). Clinic blood pressure dropped from 151.7/86.4 to 138.4/80.2 mm Hg in the intervention group after 12 months, and from 151.6/85.3 to 141.8/79.8 mm Hg in the usual care group; this gave a mean difference of −3.4 mm Hg (95% confidence interval −6.1 to −0.8) in systolic blood pressure and −0.5 mm Hg (−1.9 to 0.9) in diastolic blood pressure (table 2). The results were similar in the complete case analysis and showed a smaller but still significant effect size when considering the mean of the second to sixth blood pressure readings (table A2). Similarly, considering the primary outcome data as repeated measures over the 12 months and controlling for baseline, a significant difference remained between groups in favour of the HOME BP intervention: −2.9 mm Hg (95% confidence interval −4.8 to −1.1 mm Hg) for systolic blood pressure and −0.6 mm Hg (−1.6 to 0.5 mm Hg) for diastolic blood pressure.

**Table 2:** Mean (standard deviation) blood pressure at baseline, six months, and 12 months using second and third measurements, and adjusted difference

Blood pressure	Baseline	6 months	12 months	Imputed (100 imputations)			Complete cases	
				Adjusted difference at 6 months*	Adjusted difference at 12 months*		Adjusted difference at 6 months*	Adjusted difference at 12 months*
**Systolic blood pressure (mm Hg)†**								
Usual care	151.6 (11.1)	140.9 (16.0)	141.8 (16.8)	—	—		—	—
Intervention	151.7 (11.8)	138.7 (17.0)	138.4 (16.0)	−2.3 (−4.9 to 0.3)	−3.4 (−6.1 to −0.8)		−2.3 (−4.8 to 0.3)	−3.5 (−6.2 to −0.9)
**Diastolic blood pressure (mm Hg)**								
Usual care	85.3 (9.9)	80.2 (10.3)	79.8 (10.1)	—	—		—	—
Intervention	86.4 (9.6)	79.9 (9.7)	80.2 (10.1)	−1.0 (−2.4 to 0.4)	−0.5 (−1.9 to 0.9)		−1.0 (−2.4 to 0.3)	−0.5 (−1.8 to 0.9)

* Mean difference (95% confidence interval) controlling for baseline blood pressure, age, sex, and blood pressure target, with a random effect for practice.

† Systolic blood pressure at 12 months was the primary outcome.

A post hoc analysis showed that blood pressure dropped by at least 5 mm Hg in 201/270 (74.4%) of the intervention group compared with 170/282 (60.3%) of those receiving usual care: adjusted odds ratio for a 5 mm Hg drop: 1.9 (95% confidence interval 1.3 to 2.8; controlling for practice level clustering and stratification factors).

**Fig 2:**
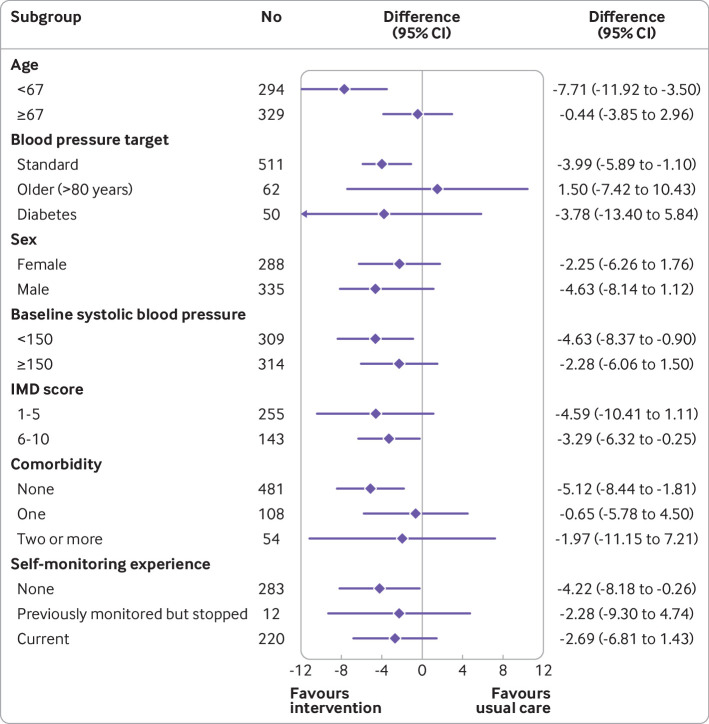
Exploratory subgroup analyses showing effect sizes in usual care and intervention groups. IMD=index of multiple deprivation (lower score means less deprived)

Exploratory subgroup analyses (fig 2, table A3) suggested that participants aged 67 or older had a smaller effect size (−0.4 mm Hg, 95% confidence interval −3.9 to 3.0 mm Hg) than those younger than 67 (−7.7 mm Hg, −11.9 to −3.5 mm Hg). Similarly, while the effect sizes in the standard and diabetes target groups were similar (−4.0 mm Hg, −6.9 to −1.1 mm Hg; −3.8 mm Hg, −13.4 to 5.8 mm Hg, respectively), those older than 80 years with a higher target of 145/85 mm Hg showed little evidence of benefit (1.5 mm Hg, −7.4 to 10.4 mm Hg). Results for other subgroups, including sex, baseline blood pressure, deprivation, and history of self-monitoring, were similar between groups; however, it is possible that those with comorbidities gained less from the intervention (systolic blood pressure difference, controlled for age—no comorbidity: −5.1 mm Hg, −8.4 to −1.8 mm Hg; one comorbidity: −0.6 mm Hg, −5.8 to 4.5 mm Hg; multiple comorbidities: −2.0 mm Hg, −11.1 to 7.2 mm Hg).

Information about possible adverse effects was derived from an extended version of the illness perceptions questionnaire symptoms section and showed no differences between groups^29^ (table 3). More participants in the intervention group reported weight loss (29/243, 11.9% *v* 57/251, 22.7%; P=0.002), but this was not borne out by the objective weight data (mean difference −0.36 kg, 95% confidence interval −1.10 to 0.38 kg; table A4).

**Table 3:** Participants who reported a hypertension drug specific symptom or adverse effect at final follow-up. Data are numbers (percentages)

Reported symptoms	Control	Intervention	P value for difference
**General symptoms**			
Stiff joints	140/252 (55.6)	138/243 (56.8)	0.92
Pain	118/252 (46.8)	116/244 (47.5)	0.40
Sleep difficulties	129/252 (51.2)	111/243 (45.7)	0.54
Fatigue	109/252 (43.3)	112/242 (46.3)	0.15
Cough	94/251 (37.5)	85/246 (34.6)	0.75
Loss of strength	62/251 (24.7)	75/244 (30.7)	0.19
Sore eyes	62/251 (24.7)	71/244 (29.1)	0.86
Pins and needles	69/250 (27.6)	71/246 (28.9)	0.92
Loss of libido	59/249 (23.7)	63/238 (26.5)	0.55
Headaches	72/251 (28.7)	64/245 (26.1)	0.96
Dry mouth	64/250 (25.6)	63/244 (25.8)	0.95
Breathlessness	50/251 (19.9)	57/244 (23.4)	0.55
Sore throat	44/250 (17.6)	51/243 (21.0)	0.20
Fast heart rate	40/251 (15.9)	41/243 (16.9)	0.52
Mood change	33/250 (13.2)	36/243 (14.8)	0.90
Wheeziness	32/251 (12.8)	32/245 (13.1)	0.77
Nausea	26/251 (10.4)	22/242 (9.1)	0.51
Rash	16/249 (6.4)	16/243 (6.6)	0.77
Other	43/240 (17.9)	35/229 (15.3)	0.23
**Hypertension specific symptoms**			
Swelling of legs or ankles	59/251 (23.5)	65/247 (26.3)	0.26
Feeling flushed	40/250 (16.0)	47/243 (19.3)	0.23
Upset stomach	45/252 (17.9)	50/242 (20.7)	0.18
Dizziness	47/251 (18.7)	40/245 (16.3)	0.64
Impotence	37/248 (14.9)	36/234 (15.4)	0.87

Participants who used the digital intervention were more likely to have their antihypertensive drugs adjusted during the study; this included more changes in dose (relative risk of a dose change, intervention *v* usual care: 2.0, 95% confidence interval 1.5 to 2.7) and more changes in drugs (relative risk of a drug change, intervention *v* usual care: 1.5, 1.1 to 1.9; table 4). Self-reported adherence in both groups was high throughout (Medication Adherence Rating Scale questionnaire^27^: median baseline 24, maximum possible 25, interquartile range 23-25; at 12 months: 24, interquartile range 23-25 in the control group, and 24, interquartile range 24-25 in the intervention group; P=0.97 for the difference).

**Table 4:** Number of drugs and dose changes in usual care and intervention groups

Drug and dose changes	Median (interquartile range)	Mean (standard deviation)	Relative risk* (95% confidence interval)
**No of any type of drug change during study period†**			
Usual care (n=293)	0 (0-1)	0.9 (1.4)	1.0
Intervention (n=283)	1 (0-2)	1.5 (1.7)	1.7 (1.4 to 2.1)
**No of dose changes**			
Usual care (n=293)	0 (0-0)	0.4 (0.7)	1.0
Intervention (n=283)	0 (0-1)	0.7 (1.1)	2.0 (1.5 to 2.7)
**No of drug changes**			
Usual care (n=293)	0 (0-1)	0.5 (1.0)	1.0
Intervention (n=283)	0 (0-1)	0.8 (1.1)	1.5 (1.1 to 1.9)

* Data follow a negative binomial distribution and analysis controls for same factors as primary analysis, and baseline number of drugs.

† Increase in dose or additional drugs.

Engagement with the digital intervention was high, with 281/305 (92%) participants completing the two core training sessions, 268/305 (88%) completing a week of practice blood pressure readings, and 243/305 (80%) completing at least three weeks of blood pressure entries (table A5). Furthermore, 214/305 (70%) were still monitoring in the last three months of participation (of 12 month study). However, less than one third of participants chose to register on one of the optional lifestyle change modules. Of the subsample of 243 participants with a body mass index greater than 25, 46 (19%) registered on the online weight loss programme.

The patient enablement score showed a reduction over time (that is, increased enablement) in the self-management group. This reduction meant that by 12 months a significant difference was found (−0.4, 95% confidence interval −0.5 to −0.2) between the usual care and intervention groups (table 5).

**Table 5:** Patient enablement in usual care and intervention groups

Group	No of participants with measures at both time points	Mean baseline PEI (standard deviation)	Mean 12 month PEI (standard deviation)	Difference in PEI at 12 months (95% confidence interval)*
Usual care	246	3.0 (1.0)	3.1 (1.1)	Reference
Intervention	252	3.1 (1.1)	2.8 (1.0)	−0.4 (−0.5 to −0.2)

Modified patient enablement instrument (PEI) is scored from 1 to 7, with lower scores implying higher enablement.^26^

* Model as per primary outcome but also controlling for baseline PEI.

After 12 months, a post hoc analysis showed that 112/234 (47%) patients in the usual care group reported monitoring their own blood pressure at home at least once per month during the trial; of these, 78 (70%) said that they took their readings to their general practitioner. Of 56/76 (74%) general practitioners who responded, 35 (63%) reported using home readings in their titration decisions for usual care.

The within trial analysis for quality of life (EuroQoL-5D-5L) showed no significant difference between the two groups (table 6). The difference in mean cost per patient was £38 ($51.3, €41.9; 95% confidence interval £27 to £47), which along with the decrease in systolic blood pressure, gave an incremental cost per mm Hg blood pressure reduction of £11 (£6 to £29; table 7). Figure A1 shows the results of bootstrapping the incremental cost and blood pressure gains, which are summarised in the cost effectiveness acceptability curves (fig A2). These curves show the intervention had high (90%) probability of being cost effective at willingness to pay above £20 per unit reduction. The probabilities of being cost effective for the intervention against usual care were 87%, 93%, and 97% at thresholds of £20, £30, and £50, respectively.

**Table 6:** Quality of life measured by using EuroQoL-5D-5L in usual care and intervention groups. Data are mean (standard deviation) unless stated otherwise

Group	Baseline	6 months	12 months	Imputed (100 imputations)			Complete cases	
				Difference in AUC at 6 months*	Difference in AUC at 12 months*		Difference in AUC at 6 months*	Difference in AUC at 12 months*
Usual care	0.90 (0.13)	0.92 (0.10)	0.90 (0.12)	—	—		—	—
Intervention	0.89 (0.14)	0.89 (0.15)	0.90 (0.14)	−0.007 (−0.02 to 0.002)	0.002 (−0.007 to 0.01)		−0.006 (−0.02 to 0.003)	0.002 (−0.008 to 0.01)

AUC=area under curve.

* Mean difference (95% confidence interval).

**Table 7:** Costs, systolic blood pressure reduction from baseline, and incremental cost per blood pressure reduction by using bootstrap methods based on imputed blood pressure data. Data are mean (95% confidence interval)

Group	Cost (£)	Incremental cost (£)	Systolic blood pressure reduction (mm Hg)	Incremental systolic blood pressure reduction (mm Hg)	ICER*
Usual care	92 (85 to 99)	^—^	9.8 (8.2 to 11.5)	—	—
Intervention	130 (122 to 137)	38 (27 to 47)	13.2 (11.7 to 14.8)	3.5 (1.3 to 5.6)	11 (6 to 29)

£1.00=$1.35, €1.10. ICER=incremental cost effectiveness ratio.

* Blood pressure reduction (£/mm Hg).

## Discussion

### Main findings

A digital intervention enabling self-management of hypertension, including self-monitoring, titration based on self-monitored blood pressure, lifestyle advice, and behavioural support for patients and healthcare professionals, resulted in a worthwhile reduction of systolic blood pressure achieved at modest cost. This finding was robust in sensitivity analyses, including complete case analysis, and also when the mean of the second to sixth blood pressure readings was used as the outcome. The reduction was achieved through increased titration of antihypertensive drugs with no increase in adverse effects, suggesting that the HOME BP digital intervention reduces clinical inertia and leads to optimisation of treatment. The effect size observed from this intervention could be expected to result in a reduction of 10-15% in patients having a stroke and a reduction of 5-10% in patients having coronary events. Given the low marginal cost, such an effect could make a major difference to the millions of people being treated for hypertension in the UK and worldwide.

### Strengths and weaknesses

This was a large trial of a digital intervention in the field of hypertension and with follow-up for a year. Adequate power existed to detect a difference in blood pressure, and over recruitment ensured such an effect was not missed. By recruiting from a large number of general practices, we ensured generalisability in terms of healthcare professionals. However, some evidence was found of preferential recruitment of those with higher socioeconomic status, although we found no evidence that socioeconomic status mediated outcomes. Although white ethnicity (94%) appears over represented in comparison to the population of England and Wales as a whole, this reflects differences in ethnicity by age: 95% of those aged 65-69 have white ethnicity.^33^

The mean age of people declining to take part was 73, and the commonest reason cited was lack of internet access, mirroring Ofcom’s latest data showing a reduction in computer access by socioeconomic status and age.^34^ While online access is increasing year on year in all age groups and societal stratums, suggesting this barrier could be reduced in the future, it will remain important to better understand barriers to uptake by those in more deprived areas.^35^ Investment in specific measures will be needed to enable the most vulnerable to engage with digital health initiatives and mitigate the risk that digital health contributes to a widening of health inequality, particularly as deprivation did not modify the effects of intervention.

The study used minimisation to reduce important baseline imbalance and this has the potential to reduce the effect of randomisation. However, we have no evidence that randomisation concealment was affected. Similarly imputation can influence results, but with high follow-up (89%) and equivalent complete case results this seems unlikely.

While prescribing records suggested an increase in drug use, and the questionnaires suggested high rates of drug adherence, data were not available about filling of prescriptions or validated adherence. The best measures of drug adherence use electronic systems, which were not available for this study. However, other work using such methods suggests that self-monitoring improves adherence.^36^ Taking into account all our observations, it appears likely that increased antihypertensive drug use drove lower blood pressure.

The effect size seen in this trial was slightly smaller than, but within the confidence intervals of, our trial that assessed a similar paper based self-management intervention in a similar population. Additionally, the upper confidence interval crosses our prespecified clinically important level of 5 mm Hg.^9^ As with our previous work, the results at 12 months showed greater divergence than at six months, which suggests that the intervention might have ongoing impact. We know that just under half of patients in the control group reported self-monitoring blood pressure during the trial, and that these records were used by their general practitioners in making treatment decisions; this could reduce the effect size, although self-monitoring outside of a more complex intervention such as HOME BP has similar efficacy to usual care.^37^ Engagement with the digital intervention was high (70% were still monitoring after nine months) and equivalent to our previous work, but the home monitoring target was not as low as that in TASMINH2 (systolic blood pressure 135 *v* 130 mm Hg) owing to changes in national guidance, and this might have reduced the effect size.^9^

The self-monitoring schedule used here was developed in our previous self-management work, but is different to that recommended in subsequent international recommendations.^9^ ^10^ ^38^ ^39^ However, the requirement for 14 readings to be taken per week is in line with these recommendations, and recording the second of two morning readings each day was originally chosen to simplify self-monitoring because morning readings are better correlated with stroke risk.^40^

Subgroup analysis suggested a differential effect by age, which is important given that such interventions have been proposed as particularly relevant to the ageing population.^4^ Participants in the younger half of the age distribution (<67 years) achieved twice the overall reduction in systolic blood pressure, and older participants (≥67 years) gained no benefit. The effect did not seem to be caused by the higher target for people older than 80 years, which has not previously been observed in other trials of self-monitoring. However, too few studies of digital interventions in hypertension have been published to assess whether it is a particular issue with the type of intervention.^7^ ^37^ Furthermore, the ageing population also influences rising levels of multimorbidity, and so the suspicion that those with comorbidities gained less from the intervention also merits further investigation.^41^ ^42^ Our process evaluation, which is published in detail elsewhere, has not found evidence of access or engagement problems, or of explanatory characteristics in older people or their clinicians, but the results might have been confounded by the differential blood pressure target for those older than 80 years (145 mm Hg).^43^ Given the inclusion criteria included a systolic blood pressure of more than 140 mm Hg, older people would not have been prompted to change drugs until their blood pressure rose 10 mm Hg higher than the younger group. Furthermore, we found some evidence of increased uptake of physical activity in the younger group, which might partly explain the findings (to be reported elsewhere).

### Relation to the literature

We observed an increase in antihypertensive drug changes in the intervention group, which suggests that the HOME BP intervention led to reduced clinical inertia. This phenomenon has been shown to result in reduced action by clinicians in the face of evidence, in this case of raised blood pressure.^44^ ^45^ Our previous work on self-monitoring and management has also resulted in increased use of antihypertensive drugs, but the data captured here are more detailed than has been previously possible.^9^ ^10^ ^46^ We used a drug titration algorithm that gave clinicians the opportunity to develop individual treatment plans for their patients, which is in line with national and international guidance.^38^ ^39^ In tandem with reduced clinical inertia, self-management in the context of HOME BP improved patient enablement, and this might also have mediated the effect. However, the behavioural aspects of the intervention were less successful and were only taken up by a small proportion of participants.

The major advantage of a digital intervention is the ability to be deployed at a low marginal cost, and the within trial cost effectiveness analysis supports this assumption. All training for the intervention was delivered online, meaning implementation can also be cost effective. This finding is reflected in the cost of £11/mm Hg reduction, which compares well with £25.66/mm Hg in the HITS trial in Scotland that used a propriety telemonitoring system.^47^ We would expect such a blood pressure reduction to lead to a longer term impact on cardiovascular events. However, to properly assess this impact on cost effectiveness in the longer term, additional modelling that takes into account these effects with extrapolation to a longer time horizon is needed, and we are in the process of such work. Previous self-monitoring interventions have proven to be cost effective in the long term within standard parameters.^11^ ^12^

Interestingly, the patient enablement instrument showed that patients were enabled to be more active in controlling their hypertension. Many chose to do this through drugs only, whereas a small number chose to include behavioural or lifestyle modifications as part of blood pressure management. The small proportions choosing behavioural support might seem counterintuitive, but as has been pointed out before, enabled patients do not always make the decisions that clinicians or public health physicians would like them to.^48^

Trials of self-monitoring blood pressure appear to work best with relatively intensive cointerventions (such as telemonitoring, educational advice, or pharmacist input) and the current study fits with that literature.^37^ In the context of the current trial, we are not able to distinguish the relative importance of the different parts of the digital intervention. Relatively few studies have been performed that combine self-monitoring with a digitally delivered cointervention, and none has shown a major effect in an adequately powered trial over a year.^7^ The HOME BP trial provides evidence that a digitally delivered intervention for hypertension can be successful over 12 months, with engagement from clinicians and patients.

### Clinical implications

Surveys suggest that most general practitioners are drawing on self-monitoring in their hypertension management, and that at least one third of patients with hypertension are self-monitoring.^49^ ^50^ Over and above the clinical benefit from the HOME BP digital intervention, the ability to manage blood pressure remotely at scale has never been so important as during the current crisis.^13^ Therefore, implementation of a cost effective digital intervention that leads to lower blood pressure would now seem to be appropriate. However, such implementation will not be possible without some consideration of the factors that influence successful translation into daily practice.^51^ Some of these factors were successfully addressed in the development of HOME BP, which used extensive user feedback to ensure that healthcare professionals and patients had a shared, positive understanding of the aims and likely benefits of HOME BP, and perceived it as easy and not onerous to use.^16^ ^17^ ^18^ ^19^ Achieving clinician buy-in is more likely to occur once evidence from trials such as ours is incorporated into routine clinical practice guidelines. A key barrier to achieving such buy-in and professional usability is probably the lack of integration of the HOME BP digital intervention into electronic health records. A system to allow deployment of proven digital interventions within the UK NHS and other health systems is now urgently needed. Examples of this are beginning to emerge; for example, general practitioners in Scotland can now get home blood pressure readings sent to their Docman electronic record system (PCTI Solutions, UK), although the underlying telemonitoring is SMS based and lacks much of the functionality included in the HOME BP digital intervention.^52^ Further work might evaluate such implementation to ensure that predicted benefits are achieved and to allow further development of the intervention, particularly for older people and those from more disadvantaged backgrounds.

## Conclusions

Overall, this digital intervention for the management of hypertension that uses self-monitored blood pressure and behavioural techniques has led to better control of blood pressure than usual care. The HOME BP digital intervention, combined with self-monitoring, has the potential to provide cost effective support for patients and professionals in lowering blood pressure. The next step is an implementation strategy to realise such benefits for the whole population.

**What is already known on this topic**

- Previous trials of self-monitoring and self-management have shown effectiveness in reducing blood pressure, but have often relied on relatively expensive technology or time consuming training packages to realise any benefit
- Short term trials of digital interventions have shown potential to improve blood pressure control, but have not provided sufficient evidence for widespread implementation

**What this study adds**

- Home and Online Management and Evaluation of Blood Pressure (HOME BP) is a digital intervention comprising self-monitoring of blood pressure with reminders and predetermined drug changes combined with lifestyle change support for the self-management of high blood pressure
- HOME BP resulted in better control of systolic blood pressure after one year than usual care and at low incremental cost; adverse events were similar to usual care
- Digital interventions such as HOME BP have the potential to be implemented at scale in a cost effective manner

## Data Availability

The HOME BP trial is a member of the BP SMART consortium of randomised controlled trials of self-monitoring in hypertension and data will be included in future meta-analyses. Anonymised trial data from HOME BP are available on reasonable request from the corresponding author.

## Web extra

Extra material supplied by authors

Web appendix: Appendix

## Notes

Contributors: LY, together with PL and RJM, conceived and led the study, providing detailed supervision of all aspects throughout. JR, EM, CRM, SM, FSM, PS, and BW provided senior expertise and leadership, contributing to designing the study, securing funding and supervising the conduct of the study. BS carried out the statistical analysis, and JR and SZ carried out the economic analysis. KM, KB, JZ, and RB developed the intervention, supported its implementation, contributed to designing the study and carried out the process evaluation. JK led trial implementation along with EO and JA, with advice from JN, and CR was the key public contributor. All authors commented on drafts of this paper. RJM will act as guarantor and affirms that the manuscript is an honest, accurate, transparent, and full account of the HOME BP trial. The corresponding author attests that all listed authors meet authorship criteria and that no others meeting the criteria have been omitted.

The lead author (RJM) affirms that the manuscript is an honest, accurate, and transparent account of the study being reported; that no important aspects of the study have been omitted; and that any discrepancies from the study as planned (and, if relevant, registered) have been explained.

Dissemination to participants and related patient and public communities: A lay summary of the results of the study will be shared with participating patients and their practices.

Provenance and peer review: Provenance and peer review: Not commissioned; externally peer reviewed.
