# Supplementary material for: Home and Online Management and Evaluation of Blood Pressure (HOME BP) using a digital intervention in poorly controlled hypertension: randomised controlled trial
Source: BMJ. 2021 Jan 19;372:m4858. doi: 10.1136/bmj.m4858 (PMC7814507; doi:10.1136/bmj.m4858)
Supplement: Supplementary file 1 — Web appendix: Appendix [file mcmr058563.ww.pdf]

Web Appendix to accompany HOME BP main trial paper

Appendix 1 Additional Tables and Figures

Table A1 *Reasons for declining screening for the trial (n=2426, mean age 73)*

	N (% of respondents who ticked this box)	Mean age of respondents who ticked this box*
I do not have access to the internet	1054 (52.0%)	76
I do not wish to take part in an internet study	720 (29.7%)	73
I do not wish to be part of a research trial	707 (29.1%)	72
I do not wish to alter my medications	432 (17.8%)	73
I do not have time to take part in the study	379 (15.6%)	66
I do not wish to measure my own blood pressure	302 (12.4%)	75
I do not want to give a reason	166 (6.8%)	70
I am unable to attend the surgery	113 (4.7%)	76
My high blood pressure is of no concern to me	59 (2.4%)	73
Other	447 (18.5%)	73

Note more than one answer possible

** in years*

Table A2 Mean blood pressure at baseline, 6 months and 12 months using measurements 2-6

				Imputed (100 imputations)		Complete cases	
Systolic blood pressure[$]	Baseline[+]	6 months[+]	12 months[+]	Adjusted* difference at 6 months	Adjusted* difference at 12 months	Adjusted* difference at 6 months	Adjusted* difference at 12 months
Usual Care	148.3 (10.8)	138.1 (14.8)	138.4 (15.4)				
Intervention	148.6 (11.8)	136.0 (15.5)	136.0 (15.3)	-2.3 (-5.0 to -0.3)	-2.6 (-5.2 to -0.1)	-2.4 (-4.7 to 0.0)	-2.6 (-5.1 to -0.1)
Diastolic blood pressure							
Usual Care	84.2 (9.6)	79.2 (10.0)	78.7 (9.9)				
Intervention	85.4 (9.6)	79.3 (9.6)	79.4 (9.8)	-0.6 (-2.0 to 0.8)	-0.4 (-1.8 to 1.0)	-0.7 (-2.0 to 0.6)	-0.6 (-1.9 to 0.7)

[+] Mean (standard deviation)

* Mean difference (95% confidence intervals) controlling for baseline blood pressure, age and BP target, with a random effect for practice

[$] Blood Pressure was measured using a BPTru 200 monitor set to record six values at 1 minute intervals.

Table A3 Sub-group analyses

	N (%)	SBP Usual care+	SBP Intervention+	Interaction term	Adjusted difference in mean SBP in subgroup (95% CI) *
**Primary outcome – mean systolic blood pressure at 12 months**					
Age				7.1 (1.7 to 12.4)	
• Below age 67 years	294 (47.2%)	144.1 (17.6)	136.4 (16.9)		-7.7 (-11.9 to -3.5)
• Age 67+ years	329 (52.8%)	140.5 (16.1)	140.6 (14.8)		-0.4 (-3.8 to 3.0)
BP target				0.9 (-3.5 to 5.4)	
• standard	511 (82.0%)	141.1 (16.5)	137.4 (15.5)		-4.0 (-6.9 to -1.1)
• older	62(10.0%)	144.8 (17.6)	146.7 (17.0)		1.5 (-7.4 to 10.4)
• diabetes	50 (8.0%)	144.7 (18.5)	141.4 (18.3)		-3.8 (-13.4 to 5.8)
Sex				-2.8 (-8.1, to 2.6)	
• Female	288 (46.2%)	141.9 (17.8)	140.1 (16.2)		-2.2 (-6.3 to 1.8)
• Male	335 (53.8%)	141.8 (15.9)	136.9 (15.7)		-4.6 (-8.1 to -1.1)
Baseline systolic BP mmHg				2.4 (-2.9 to 7.7)	
• Below 150	309 (49.6%)	139.9 (15.6)	134.9 (16.0)		-4.6 (-8.4 to -0.9)
• 150+	314 (50.4%)	143.9 (17.7)	141.7 (15.3)		-2.3 (-6.1 to 1.5)
Index of multiple deprivation				1.5 (-5.2 to 8.1)	
• 1-5	255 (64.1%)	143.0 (17.6)	138.0 (13.9)		-4.5 (-10.1 to 1.1)
• 6-10	143 (35.9%)	141.5 (16.6)	138.6 (16.6)		-3.3 (-6.3 to -0.3)

Comorbid condition				2.4 (-1.8 to 6.5)	
• None	481 (74.8%)	141.5 (16.4)	136.5 (16.1)		-5.1 (-8.4 to -1.8)
• One<br>• Multiple	108 (16.8%)<br>54 (8.4%)	142.8 (18.3)141.5 (13.5)	142.3 (14.8)<br>140 (17.5)		-0.6 (-5.8 to 4.5)<br>-2.0 (-11.1 to 7.2)
Previous experience of self-monitoring of blood pressure				0.8 (-2.2 to 3.8)	
• None	283 (45.4%)	140.5 (17.1)	136.5 (15.3)		-4.2 (-8.2 to -0.3)
• Did previously but stopped	12 (19.3%)	141.1 (19.3)	139.2 (18.3)		-2.3 (-9.3 to 4.7)
• Current	220 (35.3%)	143.5 (15.2)	140.9 (15.1)		-2.7 (-6.8 to 1.4)

SBP systolic blood pressure in mmHg

$^{+}$ Mean (standard deviation)

* Mean difference (95% confidence interval) Controlling for baseline blood pressure, age and BP target, with a random effect for practice apart from age subgroup

Table A4 Objective weight change data: Baseline and 12 months.

Group	Number with weight measures at both timepoints	Mean baseline weight kg (SD)	Mean 12 month weight kg (SD)	Difference in weight at 12 months (95% confidence interval)*
Usual care	279	83.7 (17.24)	83.3 (17.51)	
Intervention	264	85.9 (21.01)	85.1 (20.66)	-0.36 (-1.10 to 0.38)

*model as per primary outcome but also controlling for baseline weight

SD standard deviation; kg kilogram

Note: The intervention group lost a significant amount of weight during the trial (t=2.61 (263), *p*<0.05), whilst the usual care group did not (t = 1.60 (278), *p*>0.05).

Table A5. Patient engagement with the digital intervention components

**Engagement with digital intervention**	**Number (denominator)**	**% of sample**
Completion of the two core training sessions	281 (305)	92%
Completion of 1 week of practice BP readings	268 (305)	88%
Completion of three separate entries of 7 days of BP readings	243 (305)	80%
Still completing BP readings in the last 3 (of 12) months of participation	214 (305)	70%
Registration on at least one of the five lifestyle change modules*	87 (305)	29%
Number of lifestyle change modules registered for:	(305)	
One	64	21%
Two	18	6%
Three	4	1%
Four	1	<1%
Registration on online weight loss programme (This option was only offered to patients with a BMI over 25: n=243)	46 (243)	19%

*Participants could sign up to separate online interventions for each behaviour change, including standalone educational sessions regarding salt, healthy eating and alcohol with behaviour change techniques to increase self-efficacy. The physical activity intervention was an interactive online tool with tailored email prompts for increasing physical activity (Getting Active). The weight loss intervention was a complex 24-session tool (POWeR) based on self-management techniques such as goal-setting, which has been shown to be effective in a large scale RCT.[6]

Table A6 NHS Costs £/unit, 2018 prices

	By type of consultation				
**Unit costs**	**Face 2 Face**	**Telephone**	**Letter**	**Email**	**Sources**
**Primary care by staff**					
GP	34.30	8.10	1.67	1	PSSRU 2018[31]
Practice Nurse	6.45	1.52	1.67	1	PSSRU,2018
Health Care Assistant	4.00	0.94	1.67	1	PSSRU,2018
**Antihypertensive Drugs,**					
By baseline dose, by number of days	By drug				NHS Drugs Tariff,2018[30]
Changed dose, by number of days	By drug				NHS Drugs Tariff,2018
New drug, by number of days	By drug				NHS Drugs Tariff, 2018
**Hypertension related Hospital Services**					
Admission	By HRG				NHS Reference costs,2018[32]
Outpatient visit	157				NHS Reference costs,2018
A&E attendance	133				NHS Reference costs.2018
**Intervention cost per patient**					
BP Monitor Price<br>Annuitized (4 years)	£23<br><br>£6.26				
Programmer for web site	£33.46				
Total intervention cost per patient	£39.72				

Table A7 Breakdown of intervention components

Intervention component	Mode of delivery	Target	Intensity
Training	Core online module targeting outcome expectancies and self-efficacy for titrating medication and supporting patients.	Providers	Compulsory at start of trial and could be accessed freely thereafter
Training	Two core online modules, including content to increase motivation and self-efficacy to self-monitor BP, and a demonstration video via HOME BP website	Participants	Compulsory at start of trial and could be accessed freely thereafter
Self-monitoring BP	Use of automated BP monitor	Participants	7 days every 4 weeks
Entry of BP readings on website	Directly via HOME BP website	Participants	Added in one session following 7 days of recording BP in a paper diary
Automatic Feedback	HOME BP website and option to receive an email for a record	Participants	Immediately following BP entry
Automatic update on patient's average BP and any recommended actions	Email	Providers	Immediately following BP entry
Pre-planned medication titration schedule	Via medication review between prescriber and participant	Participants and providers	Following randomisation and after each set of 3 medication changes
Reminders to monitor BP	Via email	Participants	Each month
Reminders to act on raised BP	Via email	Providers	Following a raised BP reading
Lifestyle behaviours	Via HOME BP website	Participants	Optional once 9 weeks after allocation
Optional behavioural support	Via practice nurses or health care assistants	Participants	Up to six brief face-to-face, telephone or email support contacts (counted within health resource use)

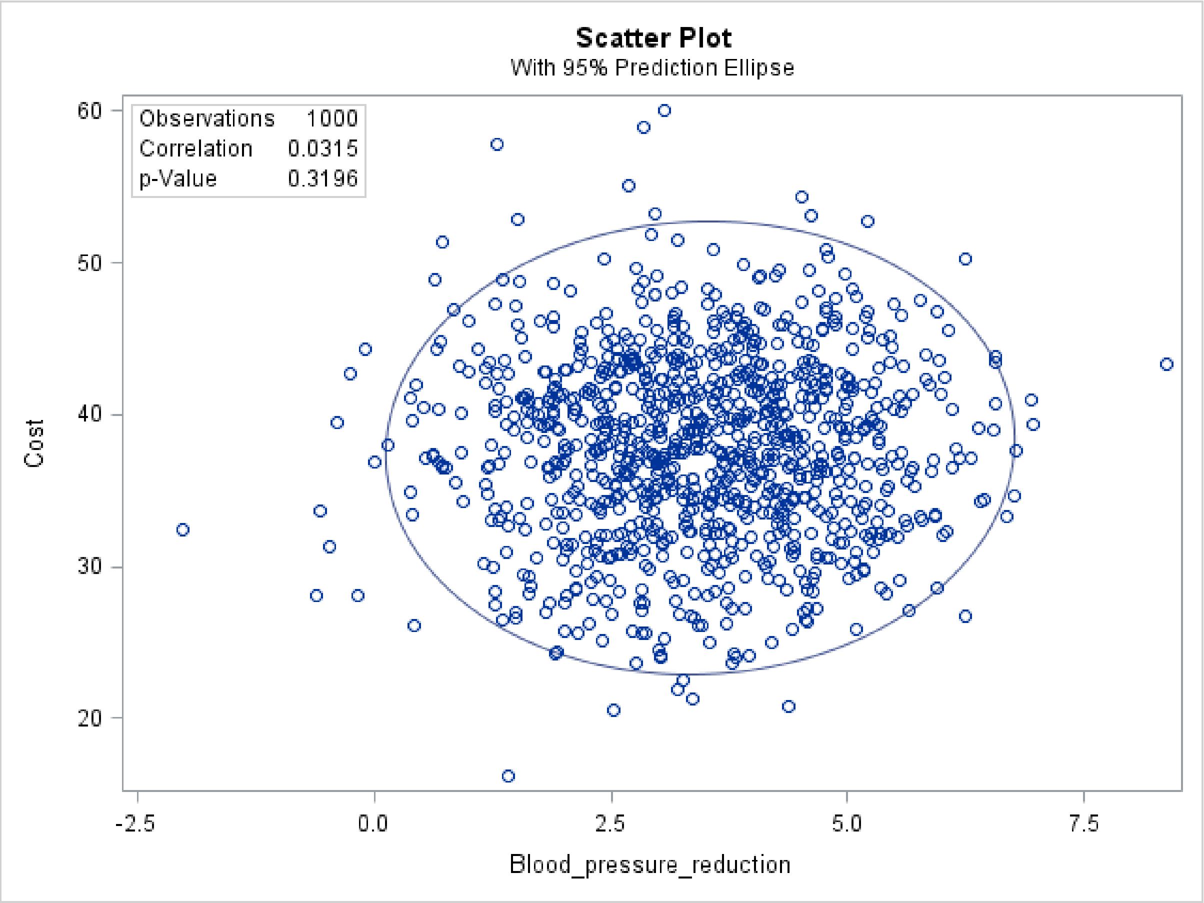

Figure A1 Scatterplot of joint distribution of incremental mean cost from NHS perspective (£s) and mean blood pressure reduction from baseline (mmHg) over 12 months

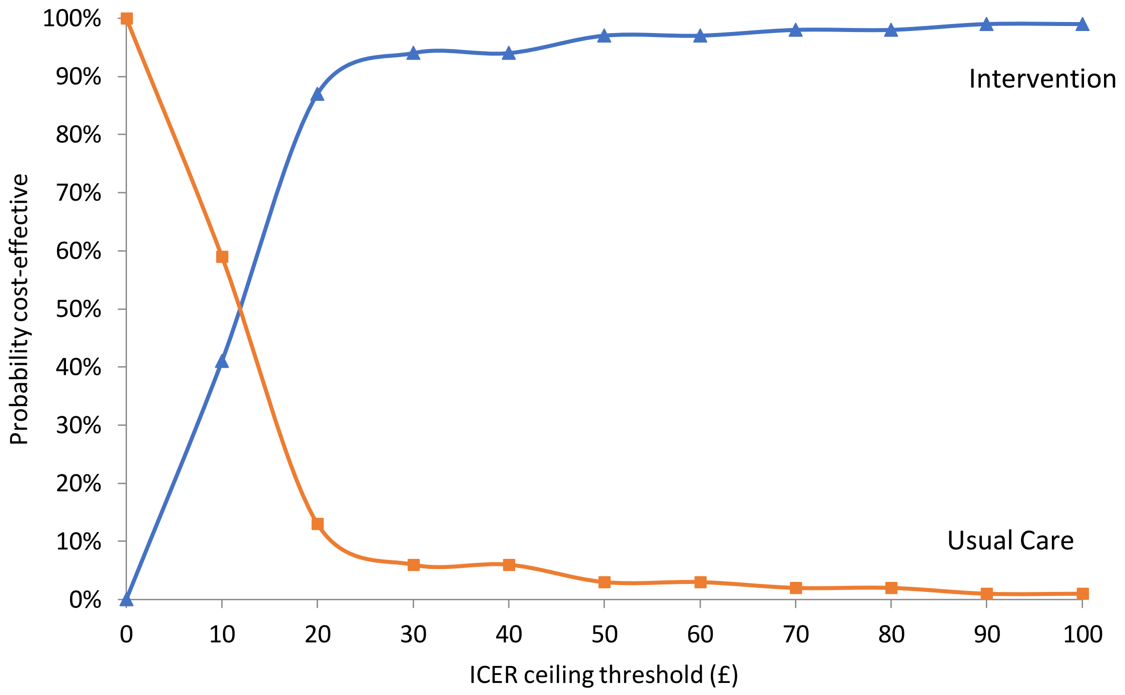

Figure A2 Cost effectiveness acceptability curve of the intervention and usual care groups based on blood pressure from baseline over 12 months.

Appendix 2 Statistical Analysis Plan

STATISTICAL ANALYSIS PLAN

Title: Home and Online Management and Evaluation of Blood Pressure (HOME BP)
Short title: HOME BP
Ethics Ref: 15/SC/0082
Trial Registration Ref: : ISRCTN13790648
Version Number and date: Version 2.1 09/05/2018

	NAME	TITLE	SIGNATURE	DATE
Written by:	Beth Stuart	Trial Statistician		18/5/18
Reviewed by:	Peter Smith	Professor of Social Statistics		22/05/2018
Approved by:	Lucy Yardley	CI		16/05/2018

<u>Version History</u>

Version:	Version Date:	Changes:
1.0	28/07/2017	

TABLE OF CONTENTS

Table of Contents 12
1 Introduction 13
1.1 Preface 13
1.2 Purpose and scope of the plan 13
1.3 Research Questions 13
1.4 Outcome measures 14
1.4.1 Primary outcome 14
1.4.2 Secondary/Tertiary outcomes 14
1.5 Sample size 15
1.6 Randomisation and blinding in the analysis stage 15
1.7 Characteristics of participants 15
1.8 Definition of population for analysis 15
2 PRIMARY ANALYSIS 16
2.1 Primary outcome 16
2.2 Handling missing and unrealistic data 16
3 SECONDARY ANALYSIS 16
3.1 Secondary outcomes 17
3.2 Tertiary outcomes 17
4 SUBGROUP ANALYSES 20
5 SENSITIVITY ANALYSES 20
5.1 Sensitivity analysis for missing data 20
5.2 sensitivity analysis for practice who diverged from protocol 20
6 SAFETY ANALYSIS 20
7 Changes to protocol or previous versions of SAP 20
8 Appendices 21

1 INTRODUCTION

1.1 PREFACE

Chief Investigator:
This SAP supports the published study protocol (http://bmjopen.bmj.com/content/bmjopen/6/11/e012684.full.pdf).

1.2 PURPOSE AND SCOPE OF THE PLAN

This document details the proposed analysis of the main paper(s) reporting results from the NIHR funded randomised controlled trial to evaluate self-management of raised BP through self-monitoring, medication adherence and lifestyle changes using the HOME BP online system with optional nurse support compared with standard care. The results reported in these papers should follow the strategy set out here. Subsequent analyses of a more exploratory nature will not be bound by this strategy, though they are expected to follow the broad principles set out here. The principles are not intended to curtail exploratory analysis (for example, to decide cut-points for categorisation of continuous variables), nor to prohibit accepted practices (for example, data transformation prior to analysis), but they are intended to establish the rules that will be followed, as closely as possible, when analysing and reporting the trial.
The analysis strategy will be available on request when the principal papers are submitted for publication in a journal. Suggestions for subsequent analyses by journal editors or referees, will be considered carefully, and carried out as far as possible in line with the principles of this analysis strategy; if reported, the source of the suggestion will be acknowledged.
Any deviations from the statistical analysis plan will be described and justified in the final report of the trial. The analysis should be carried out by an identified, appropriately qualified and experienced statistician, who should ensure the integrity of the data during their processing. Examples of such procedures include quality control and evaluation procedures.
The health economics variables, incremental QALY and cost per patient, are included in this planned within-trial analysis. These economic results will inform a long term cost effectiveness model, which is detailed elsewhere.

Trial overview
Blood pressure is a key risk factor for cardiovascular disease. The HOME BP trial aims to assess the feasibility, acceptability, effectiveness and cost-effectiveness of adding the HOME BP intervention (comprising HOME BP online digital intervention, self-monitoring, medication titration and lifestyle interventions with nurse support) into primary care for self-management of hypertension, compared to usual care.

1.3 RESEARCH QUESTIONS

Primary research question

1. Does the HOME BP intervention result in greater control of systolic blood pressure (SBP) after 1 year?

Secondary research questions

1. Does the intervention result in greater control of SBP and diastolic blood pressure (DBP) after 6 months?
2. Does the intervention result in greater control of DBP after 1 year?

1.4 Outcome measures

Baseline measures are assessed by a Practice nurse or healthcare assistant at the GP Practice. Follow-up measures are assessed at 6 and 12 months by research nurses either in the patients' own practice or at their home. Data will be collected online or on paper questionnaires, if the online data was not completed for any reason.
See Appendix II for a table of outcomes assessment schedule.

1.4.1 Primary outcome

The primary outcome is systolic blood pressure at 12 months.

Six measurements of BP are taken at the follow up visit. The mean of the 2nd and 3rd BP readings (conventional BP) will be used.

1.4.2 Secondary/Tertiary outcomes

Secondary outcomes

(i) Systolic BP at 6 months (mean of 2nd/3rd measurements – *SYSBP23*).

(ii) Diastolic blood pressure at 6 and 12 months (mean of 2nd and 3rd measurements – *DIABP23*).

(iii) The analyses will be repeated taking a repeated measures approach and exploring the effect in both systolic and diastolic BP over the 12 month period

(iv) The analyses (Systolic and diastolic BP at 6 and 12 months) will be repeated controlling for habituation. The mean of the 2-6th measurements will be used.

Tertiary outcomes

Scales are included at Appendix III.

(i) Current medications

Data on the overall number of current medications will be reported. For individual drug classes, these will be converted into defined daily doses. The DDD is the assumed average maintenance dose per day for a drug used for its main indication in adults and is defined by the WHO (http://www.whocc.no/ddd/definition_and_general_considera/).

(ii) Side effects and safety

a) **IPQ hypertension Adjusted symptoms subscale.** List of 24 symptoms with dichotomous response options of yes or no to indicate whether each symptom has been experienced in the last 4 weeks, and one 'other' option with an open-text box to clarify.

b) **Adverse events** such as admission to hospital, cardiovascular events and deaths will be recorded as part of the safety monitoring and for the economic analysis.

(iii) Quality of life

EQ5D QALY calculation by converting questionnaire scores using the standard eq5d rule set.

(iv) Resource use and costs

Cost (NHS and societal) will be estimated for each patient based on data on resources used multiplied by the relevant cost. Relevant resources include medications, those linked to adverse events (see above), and use of routine NHS services. Societal cost will comprise NHS cost plus any costs borne privately by patients. The cost of the intervention will be included for those in the intervention arm.

1.5 SAMPLE SIZE

The study requires a total sample of 244 patients per group. This allows 90% power to detect a difference in SBP of 5 mm Hg (SD 17 mm Hg) between intervention and usual care groups, based on the findings from the TASMINH-2 study. Allowing for a 15% participant drop out, we planned to recruit 287 participants per arm, resulting in a total sample size of 574 participants. Due to follow-up rates closer to 20%, the target sample size increased to 610 participants.

1.6 RANDOMISATION AND BLINDING IN THE ANALYSIS STAGE

Eligible participants were randomised in a 1:1 ratio to receive either usual care or the HOME BP intervention with optional nurse support using the HOME BP online system. Minimisation was used, taking into account participants' baseline SBP, BP target based on age (under 80/80 and over) and diabetes status, and practice. Patients were randomised to the optimal group 80% of the time. Any random numbers the minimisation routine needed were computer generated, therefore bypassing study team involvement.
The statistician will analyse the data blind to group allocation.

1.7 CHARACTERISTICS OF PARTICIPANTS

Baseline characteristics of the patients (i.e. sociodemographic data, duration of hypertension, past medical history, height and weight, blood pressure, current antihypertensive medication, adherence to medication) will be reported by randomised group.
Continuous data will be summarised in terms of the mean, standard deviation, and number of observations or, where skewed, median and lower & upper quartiles. Binary/categorical data will be summarised in terms of frequency counts and percentages.

Participant flow, from screening through randomisation, follow up and analysis will be presented in a CONSORT flow chart and include reasons for withdrawal.

1.8 DEFINITION OF POPULATION FOR ANALYSIS

All data will be included in the analysis as far as possible to allow full ITT analysis. Patients will be analysed in the groups they were allocated, irrespective of whether they received that intervention or not.

2 PRIMARY ANALYSIS

Analyses will be performed using Stata version 13 or above. All tests will be two-tailed with point estimates, 95% confidence intervals and exact p-values for the treatment effect presented.

Analyses using regression models will adjust for the factors used in minimisation (baseline SBP, and BP target based on age and diabetes status). Baseline SBP and age will be entered into the model as continuous variables. Practice was also a minimisation factor but this will be included as a random effect (see 2.1 below).
No formal adjustment for multiple significance testing will be applied. The primary approach for analysis will be with imputation of missing data to allow full ITT analysis. Patients will be analysed in the groups they were allocated, irrespective of whether they received that intervention or not.

2.1 PRIMARY OUTCOME

Descriptive summaries of mean blood pressure at baseline, 6 months and 12 months will be presented for each group.
The primary analysis will use general linear mixed modelling to compare intervention and usual care SBP at follow-up adjusting for minimisation factors and empirical confounders as defined above. The unadjusted and adjusted mean difference between groups will be presented
The assumptions of the normality of the residuals from the fixed part of the model and the normality of the random effects at the cluster level will be checked. Appropriate transformations will be considered if there is some suggestion that the assumptions for the linear model may not be met.

2.2 HANDLING MISSING AND UNREALISTIC DATA

The primary analysis for all outcomes will be with all missing values imputed using an individual chained equations multiple imputation model. This model will impute the 2nd and 3rd blood pressure measurements and then take an average of these to form a dataset with complete primary outcome measurement rather than imputing the average measurement. The model will include all blood pressure measurements taken as well as the minimisation variables and sociodemographic characteristics.
A sensitivity analysis will present the results for a complete cases analysis.
It is possible that some blood pressure values may be entered erroneously. A macro will be used to identify outliers and these values will be replaced with the average of their two immediate neighbours from the same visit. (See Appendix V)

3 SECONDARY ANALYSIS

3.1 SECONDARY OUTCOMES

Analysis of systolic BP at 6 months (mean of $2^{nd}/3^{rd}$ measurements) will be derived from the primary outcome model.
Additional blood pressure outcomes as listed below will be analysed as per the primary outcome analysis in section 4.1. Analysis of diastolic BP will adjust for baseline DBP rather than baseline SBP.

1. Systolic BP at 6 months (mean of $2^{nd}/3^{rd}$ measurements)

2. Diastolic BP at 6 and 12 months (mean of the $2^{nd}/3^{rd}$ measurements)

3. The analyses (Systolic and diastolic BP at 6 and 12 months) will be repeated controlling for habituation. The mean of the $2\text{-}6^{th}$ measurements will be used.

A secondary analysis will explore the effect over the 12 month period with readings at baseline, 6 months and 12 months using a repeated measures modelling approach. We will use a multilevel mixed model (MMLM) framework with observations (level 1) nested within participants (level 2) nested within practices (level 3). Results will be presented adjusting for minimisation variables and significant confounders as per the primary analysis.
The model will use all the observed data and makes the assumption that missing blood pressure measurements are missing at random given the observed data.

As there may not be a constant treatment effect over time, a treatment/time interaction will be modelled and included if significant, with time treated as a random effect. The model will include a random effect for practice (random intercept) and patient (random intercept and slope on time) to allow for between patient and practice differences at baseline and between patient differences in the rate of change over time (if significant), and fixed effects for baseline covariates. An unstructured covariance matrix will be used.

The assumptions of the normality of the residuals from the fixed part of the model and the normality of the random effects at the cluster level will be checked. Appropriate transformations will be considered if there is some suggestion that the assumptions for the multilevel linear model may not be met.

3.2 TERTIARY OUTCOMES

For all tertiary outcomes, linear regression will be used for continuous outcomes if the assumptions are met. Otherwise non-parametric analyses will be used. Logistic regression will be used for dichotomous outcomes and a suitable count model, as determined by goodness of fit measures, for count data. All analyses will control for stratification variables and potential confounders.
Medication defined by DDD will be analysed using mixed effect models following a similar strategy to that outlined in the analysis of the primary outcome. Where available, the baseline value will be included as a covariate along with minimisation variables.
Current medication will be analysed by drug class and overall and summarised as per the TASMIN-SR paper (table 3 below). Number of antihypertensive medications will also be analysed.

Table 3. Unadjusted Prescription of Antihypertensives (Number and Defined Daily Dose) in Intervention and Usual Care Groups[a]

	Time Point						Difference Between Intervention and Control	
	Baseline		6 Month		12 Month			
	No. of Patients	Mean (95% CI)	No. of Patients	Mean (95% CI)	No. of Patients	Mean (95% CI)	6 Month	12 Month
**No. of Antihypertensive Drugs**								
Usual care	230	1.63 (1.46 to 1.79)	226	1.75 (1.58 to 1.92)	230	1.73 (1.56 to 1.91)	0.19 (−0.01 to 0.39)	0.27 (0.07 to 0.47)
Intervention	220	1.59 (1.42 to 1.76)	215	2.07 (1.87 to 2.26)	220	2.22 (2.03 to 2.42)		
**Overall Defined Daily Dose**								
Usual care	230	2.34 (2.10 to 2.58)	226	2.57 (2.33 to 2.81)	230	2.61 (2.37 to 2.85)	0.66 (0.17 to 1.15)	0.91 (0.42 to 1.40)
Intervention	220	2.16 (1.91 to 2.40)	215	3.05 (2.80 to 3.30)	220	3.34 (3.09 to 3.59)		
**Defined Daily Dose Thiazides**								
Usual care	230	0.23 (0.17 to 0.29)	226	0.24 (0.18 to 0.30)	230	0.23 (0.17 to 0.29)	0.11 (0.02 to 0.24)	0.16 (0.04 to 0.29)
Intervention	220	0.23 (0.17 to 0.30)	215	0.35 (0.29 to 0.42)	220	0.39 (0.33 to 0.46)		
**Defined Daily Dose Calcium Channel Blockers**								
Usual care	230	0.43 (0.33 to 0.53)	226	0.52 (0.42 to 0.62)	230	0.55 (0.44 to 0.65)	0.23 (0.03 to 0.44)	0.28 (0.08 to 0.49)
Intervention	220	0.46 (0.36 to 0.57)	215	0.79 (0.68 to 0.89)	220	0.86 (0.75 to 0.96)		
**Defined Daily Dose Angiotensin-Converting Enzyme Inhibitor/Angiotensin II Receptor Blockers**								
Control		1.42 (1.24 to 1.60)	226	1.55 (1.37 to 1.73)	230	1.59 (1.41 to 1.77)	0.26 (−0.11 to 0.62)	0.34 (−0.02 to 0.70)
Intervention		1.22 (1.04 to 1.41)	215	1.61 (1.43 to 1.80)	220	1.74 (1.55 to 1.92)		
**Defined Daily Dose β-Blockers**								
Usual care	230	0.15 (0.11 to 0.19)	226	0.15 (0.11 to 0.19)	230	0.14 (0.10 to 0.18)	0.03 (−0.05 to 0.11)	0.02 (−0.06 to 0.09)
Intervention	220	0.14 (0.10 to 0.18)	215	0.17 (0.13 to 0.21	220	0.15 (0.11 to 0.19)		

[a] Defined daily dose as classified by World Health Organization. Figures combine standardized "average maintenance dose" and number of medications.[28]

Adverse events
The number and percent of people experiencing each symptom from the 12 month questionnaire will be tabulated as per Table 4 of the TASMIN-SR study (McManus et. al JAMA 2014) in order from the most commonly reported to least commonly reported. All symptoms will be reported in the statistical report, not just the top 10.
Hypoglycaemic episodes for diabetic patients in last 6 months will also be reported (recorded at 6m and 12m FU)

Table 4. The 10 Most Frequently Reported Adverse Effects Plus Selected Hypertension Medication-Specific Symptoms or Adverse Effects at 12 Months

	No. (%) of Patients		
	Usual Care (n = 230)	Intervention (n = 220)	*P* Value
Stiff joints	110 (48)	109 (50)	.72
Pain	113 (49)	101 (46)	.49
Fatigue	106 (46)	93 (42)	.42
Swelling of legs and ankles	78 (34)	81 (37)	.52
Sleep difficulties	86 (37)	71 (32)	.26
Breathlessness	66 (29)	68 (31)	.61
Dry mouth	74 (32)	58 (26)	.18
Cough	65 (28)	64 (29)	.85
Pins and needles	61 (27)	52 (24)	.48
Loss of libido	49 (21)	48 (22)	.90
Additional hypertension medication specific symptoms			
Dizziness	43 (19	53 (24)	.16
Impotence	36 (16)	37 (17)	.74
Rash	23 (10)	18 (8)	.50

Comparisons between groups will be conducted at 6 months and at 12 months using a generalised linear mixed effects model for binary data, adjusted for baseline values (symptom experienced at baseline Y/N), with a random effect for practice.
Estimating QALYs from EQ5D5L data
To derive a quality of life value at each time point based on the EQ5D5l requires a weighted average of these five values. The weights, based on a survey of public values are provided on the Health Economics journal website (https://onlinelibrary.wiley.com/doi/full/10.1002/hec.3564. Supporting Information files hec3564-sup-0001-supplementary.zip application/x-zip-compressed) The value for each patient at each time point will be a value between 0 and 1.
Any missing EQ5D5L values will be imputed using the same chained equations approach as the primary outcome.
QALYs will be analysed using an area under the curve approach. For each patient the relevant formula is QALY=(Q2-Q1)/2*T, where Q1 is initial quality of life, Q2 is the end of period quality of life and T is time. If related deaths occur and are to be included, Q2 would take the value 0. The relevant time might be the point of death if known.
Cost
Cost will be estimated by multiplying the units of service used by the price. The health economist will provide a relevant look up table for NHS costs.
Costs per unit change in the primary outcome will be calculated and reported for each trial arm.

4 SUBGROUP ANALYSES

Although we are not powered to look at subgroups, we will explore the impact of key subgroups that could plausibly modify intervention effectiveness:

- (i) Age (two categories split at median)
- (ii) BP target (standard hypertension/older hypertension/diabetes)
- (iii) Sex (Male/Female)
- (iv) Baseline BP (two categories split at median)
- (v) IMD (two categories split at median)
- (vi) Comorbidity (Three categories – none, one, multiple)
- (vii) Number of medications
- (viii) Previous experience of self-monitoring at baseline (None, Did previously but stopped, Current)

For this exploratory analysis, estimates of the interaction between subgroup and intervention will be provided with 95% confidence intervals and the estimates of the intervention effect when the subgroup is selected. Clear evidence of benefit in a subgroup will require the interaction term for that subgroup to be significant at the 5% level. Although a formal adjustment for multiplicity will not be carried out, the results will be interpreted cautiously, as at least 1 significant result might be expected by chance

5 SENSITIVITY ANALYSES

5.1 SENSITIVITY ANALYSIS FOR MISSING DATA

A complete cases analysis will be carried out to explore if the findings from this sensitivity analysis are similar to the main analysis.

5.2 SENSITIVITY ANALYSIS FOR PRACTICE WHO DIVERGED FROM PROTOCOL

One practice diverged from protocol as the nurse prescriber decided to see all usual care patients at regular intervals to monitor their blood pressure. A sensitivity analysis will be undertaken excluding all participants recruited from this practice to explore whether there is any impact on inferences from the primary analysis.

6 SAFETY ANALYSIS

Serious adverse events for the full population will be summarised descriptively according to randomised group. No statistical comparisons will be undertaken on these data

7 CHANGES TO PROTOCOL OR PREVIOUS VERSIONS OF SAP

All changes from the protocol or from previous versions of the stats plan will be detailed in the report.

8 APPENDICES

Appendix I. Schedule of study procedures

	Time point			
Measure	Baseline/ Screening	Visit 1	Visit 2	Within the HOME-BP programme
**Month**	0	6	12	(0-12)
Patient socio-demographic measures	X			
HCP demographic measures	X			
Clinical measures				
Systolic BP (SBP)	X	X	X	
Diastolic BP (DBP)	X	X	X	
Weight (kg)	X		X	
Height (cm)	X			
Diabetes status	X			
Hypoglycaemic episodes in last 6 months	X	X	X	
Medication changes (prescriptions issued)			X(NR)	
Consultations			X(NR)	
Patient self-report measures				
Patient Enablement Instrument	X		X	
Patient self-efficacy	X	X	X	
Beliefs about medication (BMQ)	X	X	X	
Medication adherence (MARS)	X		X	
Frequency of home BP monitoring (usual care group)			X	
Lifestyle behaviour changes			X	
Patient objectively recorded measures				
Website usage				X*
Usage of hypertension pages				X*
Monitoring of blood pressure				
Self-reported SBP				X*
Self-reported DBP				X*
Medication titration recommended				X*
Titration uptake				X*
Reasons for non-titration				X*
Usage of lifestyle pages				X*
Choice of lifestyle changes				X*
Reported progress on lifestyle change (e.g. weight change)				X*
HCP objectively recorded measures				
Usage of training pages (prescriber guide)				X
Emails sent to the patient				X
Titration procedure compliance				X
Support provision				X
HCP self-report measures				
Self-efficacy and outcome expectations	X_1			
Confidence in the acceptability of the intervention (for patients)	X_1			

Economic measures				
Patient quality of life (EQ-5D)	X	X	X	
Patient side effects (IPQ hypertension: Adjusted symptoms subscale)	X		X	
Costs of equipment and drugs			X(NR)	
Health professional time			X(NR)	
Patient time			X(NR)	
Qualitative process analysis				
Patient experience and views of the DI				X
HCP experience and views of the DI				X

Appendix II. Outcome assessment schedule

	Baseline	6 months	12 months
Blood pressure (sitting)	x	x	x
Blood pressure (standing)	x		
Demographics	x		
Duration of hypertension	x		
Past/new medical history	x		x
Height	x		
Current antihypertensive medications	x		x
Weight	x		x
Symptoms part of illness perception questionnaire	x		x
EQ-5D 5L	x	x	x
Lifestyle change self-report questions			x
Loss of follow-up/withdrawal		x	x
Side effects and safety			x
